# Evolutionary transition of *doublesex* regulation from sex-specific splicing to male-specific transcription in termites

**DOI:** 10.1038/s41598-021-95423-7

**Published:** 2021-08-06

**Authors:** Satoshi Miyazaki, Kokuto Fujiwara, Keima Kai, Yudai Masuoka, Hiroki Gotoh, Teruyuki Niimi, Yoshinobu Hayashi, Shuji Shigenobu, Kiyoto Maekawa

**Affiliations:** 1 Graduate School of Agriculture, Tamagawa University, Machida, Tokyo 194-8610 Japan; 2 Graduate School of Science and Engineering, University of Toyama, Gofuku, Toyama 930-8555 Japan; 3 Institute of Agrobiological Sciences, NARO (National Agriculture and Food Research Organization), Tsukuba, Ibaraki 305-8634 Japan; 4 Department of Biological Science, Faculty of Science, Shizuoka University, Suruga-ku, Shizuoka, 422-8529 Japan; 5 Division of Evolutionary Developmental Biology, National Institute for Basic Biology, Okazaki, Aichi 444-8585 Japan; 6 Department of Basic Biology, School of Life Science, The Graduate University for Advanced Studies, SOKENDAI, Okazaki, Aichi 444-8585 Japan; 7 Department of Biology, Keio University, Yokohama, Kanagawa 223-8521 Japan; 8 NIBB Research Core Facilities, National Institute for Basic Biology, Okazaki, Aichi 444-8585 Japan; 9 Faculty of Science, Academic Assembly, University of Toyama, Gofuku, Toyama 930-8555 Japan

**Keywords:** Evolutionary developmental biology, Social evolution, Entomology

## Abstract

The sex determination gene *doublesex* (*dsx*) encodes a transcription factor with two domains, oligomerization domain 1 (OD1) and OD2, and is present throughout insects. Sex-specific Dsx splicing isoforms regulate the transcription of target genes and trigger sex differentiation in all Holometabola examined to date. However, in some hemimetabolous insects, *dsx* is not spliced sexually and its sequence is less conserved. Here, to elucidate evolutionary changes in *dsx* in domain organisation and regulation in termites, we searched genome and/or transcriptome databases for the *dsx* OD1 and OD2 in seven termite species and their sister group (*Cryptocercus* woodroaches). Molecular phylogenetic and synteny analyses identified OD1 sequences of termites and *C*. *punctulatus* that clustered with *dsx* of Holometabola and regarded them as *dsx* orthologues. The *Cryptocercus dsx* orthologue containing OD2 was spliced sexually, as previously shown in other insects. However, OD2 was not found in all termite *dsx* orthologues. These orthologues were encoded by a single exon in three termites for which genome information is available; they were not alternatively spliced but transcribed in a male-specific manner in two examined species. Evolution of *dsx* regulation from sex-specific splicing to male-specific transcription may have occurred at an early stage of social evolution in termites.

## Introduction

In insects, sex determination is cell-autonomously controlled by a cascade composed of several genes, where the *doublesex* (*dsx*) encoding sex-specific transcription factors is located at the base^1^. In the cascade of the fruit fly (*Drosophila melanogaster*), the number of X chromosomes is the primary signal for sex determination^2^ and is sequentially transduced into the sex-specific splicing of *Sex-lethal*, *transformer*, and *dsx*, resulting in the sex-specific transcription of target genes responsible for their sex differentiation^3,4^. Although insect taxa differ in upstream genes encoding splicing regulators in this cascade, *dsx* is constantly present among them as well as in some crustaceans and chelicerates that are non-insect arthropods^1,5–7^. Moreover, the *dsx* orthologue is sexually spliced in all four major holometabolous insect orders examined to date (Coleoptera, Hymenoptera, Lepidoptera, and Diptera)^3,4^; thus, such *dsx* regulation is believed to be a general feature of the cascade in all insects. However, in hemimetabolous insects, *dsx* is sexually spliced in two of four species recently examined, but is spliced alternatively and not sex-specifically in the others^8,9^. Furthermore, in some crustaceans and mites, *dsx* does not produce sex-specific splicing isoforms and is instead expressed in a sex-specific manner; this controls their male differentiation^5,7^. Therefore, in contrast to knowledge based on holometabolous insects, the sex-specific regulation of *dsx* may be evolutionarily labile among insects and non-insect arthropods and could have diversified in hemimetabolous insects.

A recent study by Price et al.^6^ contributed a great deal of understanding to *dsx* evolutionary history. They searched public databases of insect genomes and transcriptomes for *dsx* homologues based on the presence of two domains conserved throughout holometabolous insects: the DNA-binding DM domain and the dimerization domain, referred to as oligomerization domain (OD) 1 and OD2, respectively. OD1 is found not only among *dsx* but also *doublesex/mab-3 related transcription factor* (*DMRT*) genes that are present among metazoans and involved in their sexual development^10,11^, whereas OD2 is specific to *dsx*^12^. They failed to obtain any evidence of the presence of *dsx* in several hemimetabolous insects, including termites (infraorder Isoptera or epifamily Termitoidae), a monophyletic group within the cockroaches (Blattodea)^13,14^. In the lower termite *Zootermopsis nevadensis*, neither domain was found, and only OD1 was found in the higher termite *Nasutitermes takasagoensis*^6^. A *dsx* homologue has been identified in the genome of the German cockroach *Blattella germanica* (gene ID: PSN43312.1^15^). *Blattella dsx* was shown to contain both OD1 and OD2 (referred to as “DM domain” and “Dsx Dimerization domain”, respectively, in Wexler et al.^9^) and to be alternatively spliced in a sex-specific manner as in holometabolous insects^9^. The rapid sequence divergence of *dsx* (or part of it) might be the reason why the orthologues could not be found in termites. We hypothesise that the *dsx* sequence (especially OD1 and/or OD2) diversified during termite evolution, and *dsx* sequence diversification subsequently affected its regulation. These hypotheses can be examined using a comprehensive search of complete genome sequences and transcriptomic data in termites and in their sister group, subsocial woodroaches (*Cryptocercus* spp.)^16–22^.

The present study aims to elucidate evolutionary changes in *dsx* in both domain organisation and its manner of regulation in the cockroach and termite clade. First, we exhaustively searched genome and transcriptome databases of the subsocial woodroach *Cryptocercus punctulatus* and seven termite species (*Hodotermopsis sjostedti*, *Cryptotermes secundus, Reticulitermes speratus*, *Coptotermes formosanus*, and *Macrotermes natalensis*, as well as *Z. nevadensis* and *N. takasagoensis*) for the OD1 and OD2 of *dsx*, and then examined whether the searched sequences were *dsx* homologues based on molecular phylogeny and synteny. Second, we performed gene expression analysis based on quantitative RT-PCR and published transcriptome data, for one subsocial woodroach and two termite species. Third, to identify putative regulatory factors in the sex-specific transcription of termite *dsx*, transcription factor binding sites (TFBSs) were identified in the promoter region of the *dsx* orthologue of *R. speratus*. The expression levels of putative transcription factors were compared between the sexes using published transcriptome data*.* Based on the results obtained, we conclude that *dsx* regulation shifted from sex-specific splicing to male-specific transcription at an early stage of social evolution in termites.

## Results

### The search for *doublesex* orthologues in termites and a subsocial woodroach

**Figure 1:**
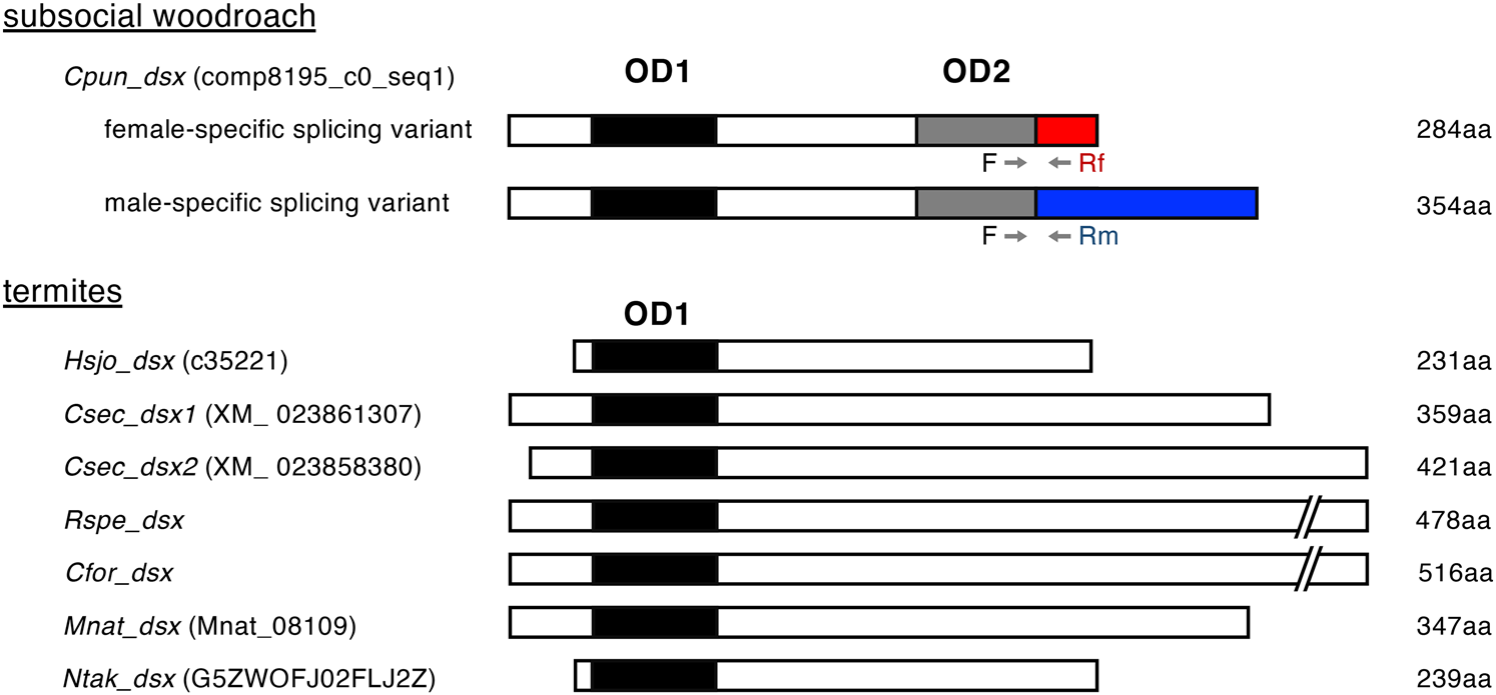
Domain organisations of *dsx* transcripts in a subsocial woodroach and in termites. Full-length coding sequence of *dsx* transcripts in one woodroach and six termite species are shown as transcribed from left to right. The OD1 is shown as a filled box, whereas the sexually shared, female-specific, and male-specific OD2 is shown as a grey, red, and blue box, respectively. Numbers on the right of boxes are amino acid lengths of the translated *dsx*. Positions of primers for quantifying *Cpun_dsx* expression by quantitative RT-PCR are shown in arrows: “F”, “Rf”, and “Rm” indicate the shared forward primer, reverse primer for the female-specific splicing variant, and reverse primer for the male-specific variant, respectively (Table S2).

We performed BLAST searches using the translated OD1 sequence of *Blattella dsx* (45 amino acids) as a query against genome and/or transcriptome databases of seven termite species and one *Cryptocercus* woodroach species, after which two to four OD1-containing sequences were hit in each species (Table 1). BLAST searches were then performed using the translated OD2 sequence of *Blattella dsx* (45 amino acids) as a query. A single sequence from the woodroach was hit (Cpun_comp8195_c0_seq1, Table 1), but no sequence was hit from any tested termites. Rapid amplification cDNA ends (RACE) PCR was performed using primers specific for Cpun_comp8195_c0_seq1 (Table S1), and a single full-length transcript was obtained only from females. Then, using a reverse primer specific to the male-specific exon of *Blattella dsx* (located at terminal codon and 3′UTR, Table S1), another 3′ end of coding sequence was amplified from males. As these transcripts contained an OD1 upstream of OD2 (Fig. 1), it was designated as *C. punctulatus dsx* (*Cpun_dsx*). The amplified sequences downstream of OD2 were predicted to be the sex-specific exon(s) of *Cpun_dsx*. The determined nucleotide and putative amino acid sequences of the *Cpun_dsx* splicing variants are available in the DDBJ/EMBL/GenBank databases (accession no. LC635715 and LC635716).

**Table 1:** Results of BLAST search for OD1 against the genomes and/or transcriptomes of seven termite species and their sister group (subsocial woodroach). Bold italic letters are gene names used in this study.

	Database	*dsx*	*Dmrt11*	*Dmrt93*	*Dmrt99*
*Zootermopsis nevadensis*	Genome (Znev OGS v2.229)	No hit	scaffold668	Znev05388^a^	Znev16235^a^
*Hodotermopsis sjostedti*	Transcriptome (DRA000538 and DRA001044)	***Hsjo_dsx*** (c35221, LC635719)	c18070	c38968	No hit
*Cryptotermes secundus*	Genome (Csec_1.0), Transcriptome (PRJNA382129)	***Csec_dsx1*** (XM_023861307), ***Csec_dsx2*** (XM_023858380)	XM0238633381	XM0238639211	XM0238500541
*Reticulitermes speratus*	Genome (Rspe OGS1.0), transcriptome (DRA010978)	***Rspe_dsx*** (scaffold6: 6307974..6309410, LC635717)	RS007930	RS006912	RS002870
*Coptotermes formosanus*	Genome (CopFor1.0)	***Cfor_dsx*** (scaffold506: 427884..429431)	GFG33987	Scaffold9383	GFG38119
*Macrotermes natalensis*	Genome (Mnat_gene_v1.2.cds), transcriptome (PRJNA382034)	***Mnat_dsx*** (Mnat_08109)	No hit	Mnat_01812	Mnat_08410
*Nasutitermes takasagoensis*	Transcriptome (DRA001046)	***Ntak_dsx*** (G5ZWOJF02FLJ2Z^a^, LC635718)	comp25194	comp174542	No hit
*Cryptocercus punctulatus*	Transcriptome (PRJDB4695)	***Cpun_dsx***^b^ (Cpun_comp8195_c0_seq1, LC635715, LC635716)	comp1991	No hit	No hit

^a^Previously described by Price et al.^6^.

^b^Found by BLAST search using OD2 (but not OD1) sequences of *Blattella dsx* orthologues, followed by 5′ RACE.

**Figure 2:**
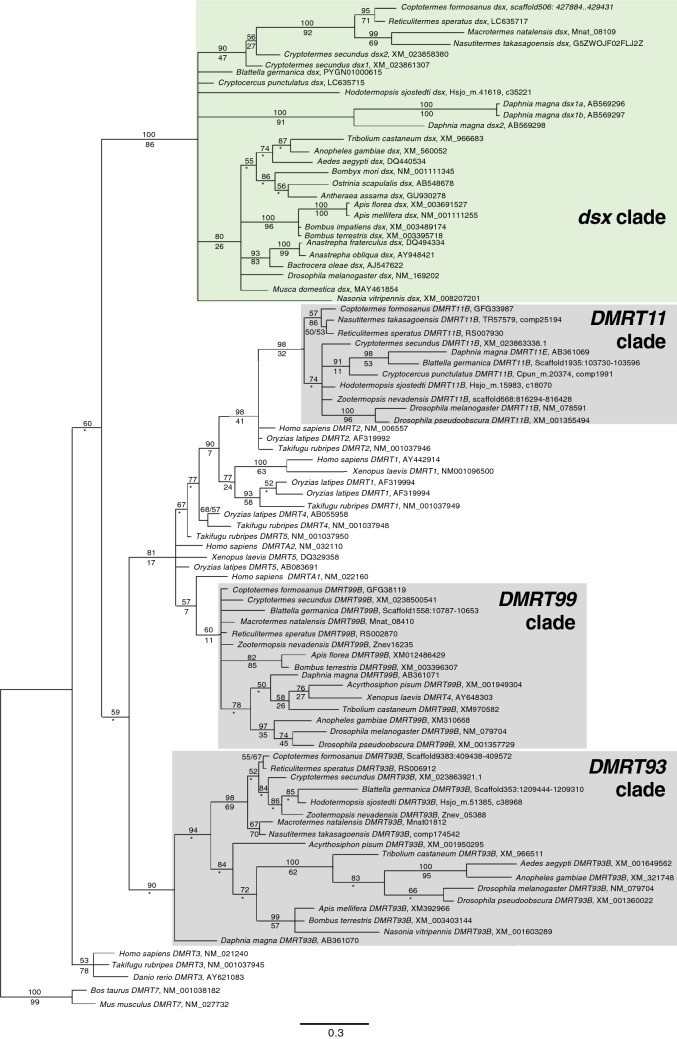
Molecular phylogenetic tree of *dsx* and *DMRT* homologues. Bayesian tree of *dsx* and *DMRT* of insects and crustaceans was constructed based on the OD1 sequences (135 bp with no gaps). Numbers shown above branches represent the Bayes posterior probabilities. Bootstrap values (1000 replicates) are shown below branches to indicate the level of support in the ML method. An asterisk indicates that a node is not supported by the ML method.

To examine the orthology of *dsx* in insects, we performed a phylogenetic analysis using OD1 nucleotide sequences searched in termites and a *Cryptocercus* woodroach, as well as those of *dsx* and *Dmrt* orthologues in other species. The phylogenetic analysis revealed that all *dsx* reported in holometabolous insects, the German cockroach, and a water flea belonged to a single clade (*dsx* clade) (Fig. 2). This monophyletic *dsx* clade also contained a single OD1 sequence from each of the four termites *H. sjostedti* (c35221^16^), *R. speratus* (not annotated, scaffold 6^20^: 6307974.0.6309410), *Coptotermes formosanus* (not annotated, scaffold 506^22^: 427884.0.429431), *N. takasagoensis* (G5ZWOJF02FLJ2Z^16^), and *M. natalensis* (Mnat_08109^18^), and two sequences from *Cryptotermes secundus* (XM_023861307 and XM_023858380^15^) (Fig. 2, Table 1). We confirmed that the sequences containing these *dsx*-clade OD1 did not possess a OD2 sequence that was unlike *Cpun_dsx*. No *dsx-*clade OD1 sequence from *Z. nevadensis* was identified in this phylogenetic analysis. The other OD1 sequences from insects formed clades specific to the *DMRT11*, *DMRT93,* and *DMRT99* orthologues (Fig. 2).

To confirm whether the *dsx*-clade sequences in termites were *dsx* orthologues, synteny analyses for *dsx* were conducted based on the proximity of the *dsx* and *prospero* (*pros*), which is well conserved among eight different holometabolous and hemimetabolous insect orders^9,23^. The gene encoding the *dsx*-clade sequence was located 83 kb, 29 kb, and 80 kb from the *pros* orthologues (RS012493^24^, GFG36637^22^, and MN005277^25^) in scaffold 6 of *R. speratus*^20^, scaffold 506 of *Coptotermes formosanus*^22^ and scaffold 295 of *M. natalensis* (Mnat_08109), respectively. Therefore, these *dsx-*clade genes were designated as the termite orthologues of *dsx* (*Rspe_dsx*, *Cfor_dsx*, and *Mnat_dsx*, Table 1). In *C. secundus*, both *dsx*-clade sequences, XM_023861307 and XM_023858380, and the *pros* orthologue (XM_023851644) were located in scaffold 829 (length: 88.5 kb), 635 (length: 1.3 Mb), and 511 (length: 2.2 Mb), respectively. Because the scaffold lengths (especially for scaffold 829) appeared insufficient for the synteny analysis based on proximity (17–245 kb^9^), evidence of synteny in *C. secundus* could not be obtained. The synteny in *H. sjostedti* and *N. takasagoensis* could not be examined because their genome data are unavailable (as of 12 July 2021). Based on OD1 sequence similarity (Fig. 2), however, we designated these as *dsx* orthologues (hereafter referred to as *Csec_dsx1*, *Csec_dsx2*, *Hsjo_dsx*, and *Ntak_dsx*, Table 1).

### Splicing patterns and expression of *doublesex* homologues in termites and a subsocial woodroach

**Figure 3:**
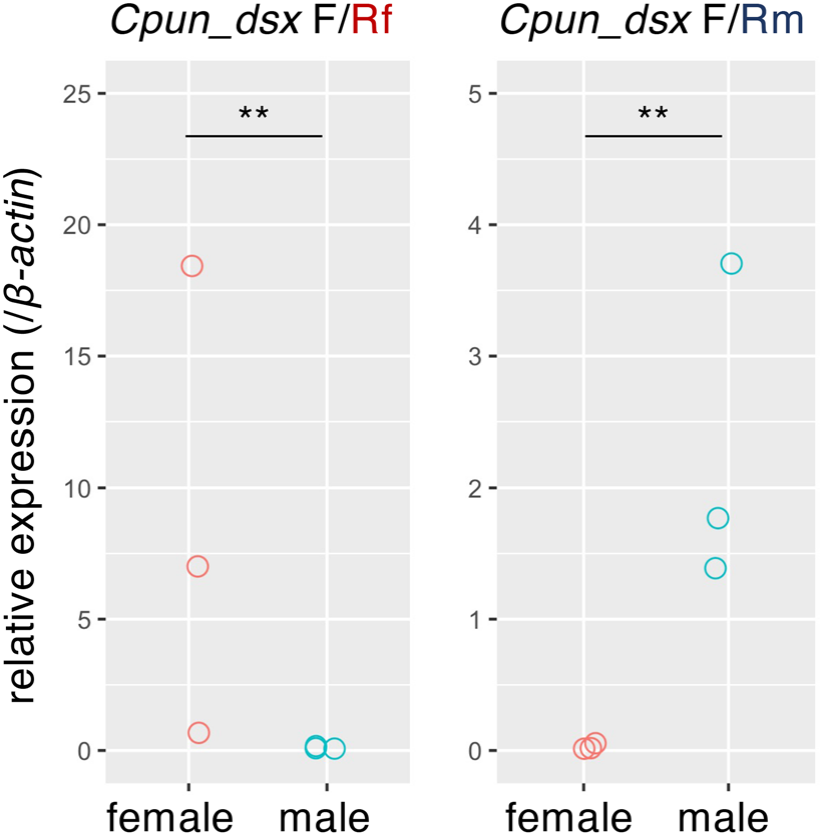
Expression patterns of *Cpun_dsx* in the female and male gonads of *C. punctulatus* adults. Left and right panels show the relative expression levels of its female- and male-specific isoform to those of *β-actin*, respectively. “F”, “Rf”, and “Rm” indicate the primers shown in Fig. 1. Replications of gonads are derived from individuals collected from three different kin groups. The expression levels were compared between sexes using GLM analyses. ** indicates *p* < 0.01.

**Figure 4:**
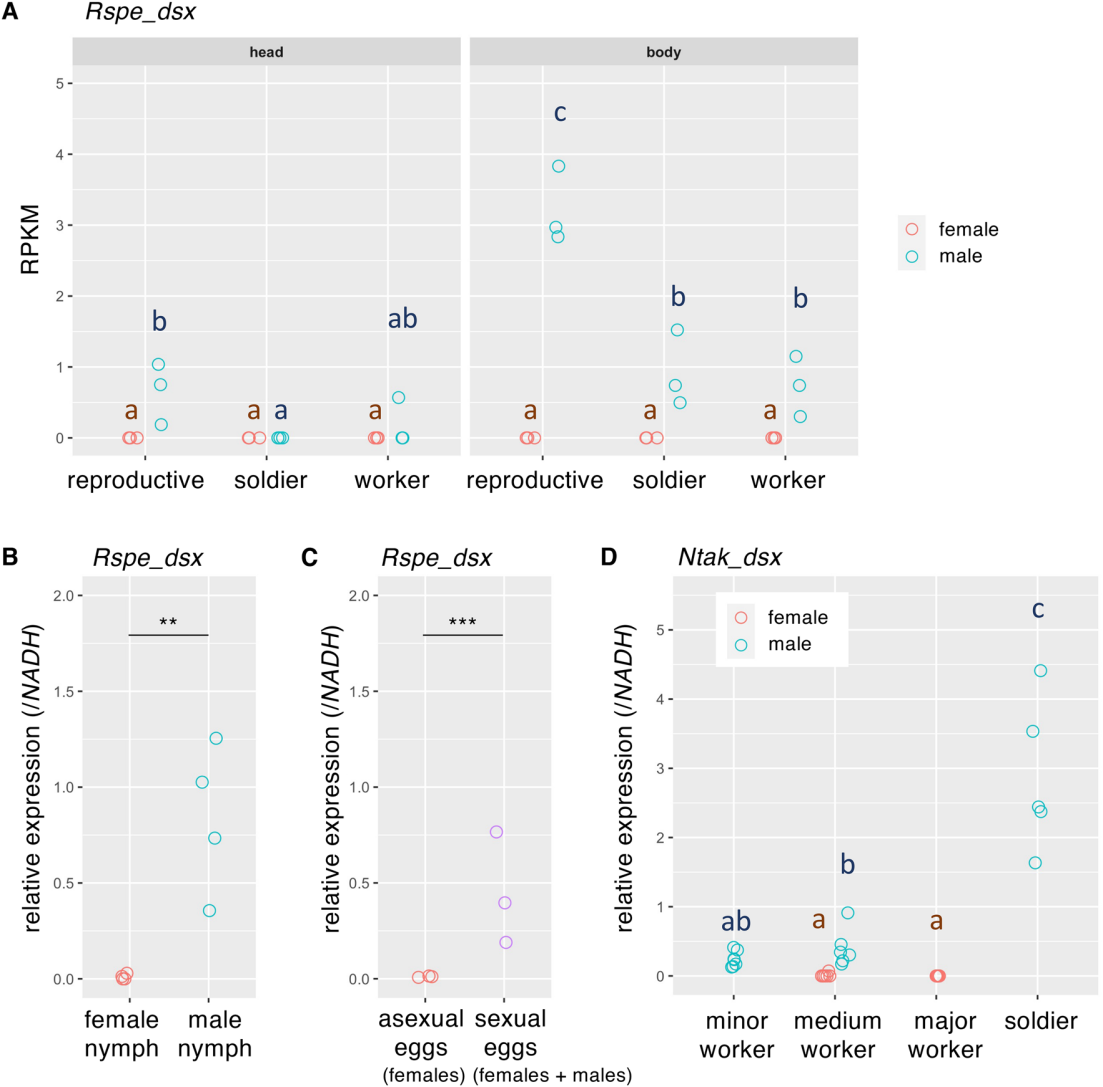
Sex-specific expression patterns of *Rspe_dsx* and *Ntak_dsx*. (**A**) Reads per kilo base per million mapped reads (RPKM) of *Rspe_dsx* in each sex and in each caste of *R. speratus*, which were re-analysed RNA-seq data (DRA010978, biological triplicates^21^). (**B**) *Rspe_dsx* expression levels relative to those of *NADH* in female and male nymphs. All nymphs examined were collected from a single colony (i.e., siblings). (**C**) Relative *Rspe_dsx* expression in eggs produced asexually and sexually. The former are fated to develop into females, whereas the sexual fate of the latter was not determined. Total RNA was extracted from 30 eggs, and three RNA samples were derived from different colonies. (**D**) *Ntak_dsx* expression levels relative to those of *NADH* in each sex and in each caste of *N. takasagoensis*. All castes examined were collected from a single colony (i.e., siblings). In (**A**) and (**D**), the effects of sex, caste, and their interaction on gene expression levels were evaluated using GLM followed by multiple comparison using linear hypothesis testing with Tukey adjustment in the “multcomp” R package. Different letters indicate significant differences according to multiple comparison (*p* < 0.05). In (**B**) and (**C**), relative expression levels of *Rspe_dsx* were compared between sexes using GLM analyses. ** and *** indicates *p* < 0.01 and 0.001 in GLM, respectively.

To confirm the sex-specific splicing of *Cpun_dsx*, expression levels of the predicted sex-specific splicing isoforms were quantified by RT-PCR using primers specific to the predicted isoforms (Fig. 1 and Table S2). The predicted female- and male-specific isoforms was confirmed to be abundantly expressed in females (*t* = − 6.68, *p* < 0.01, generalised linear model [GLM] ) and males (*t* = 7.88, *p* < 0.01, GLM), respectively (Fig. 3), indicating that *Cpun_dsx* was spliced in a sex-specific manner, as shown in holometabolous insects and the German cockroach *B. germanica*^9,15^. Next, to obtain the splicing isoforms of *Hsjo_dsx*, *Rspe_dsx*, and *Ntak_dsx*, we performed 3′-RACE PCRs using gene-specific primers (Table S2) and amplified only the single fragment downstream of OD1 for each species (Fig. 1). The amplified downstream sequences of each species (accession no. LC635717–9) were consistent with those obtained by the aforementioned BLAST search. Although *dsx* is composed of ca. five exons, of which two to three posterior exons are sexually spliced in almost all insects examined previously^26^, each of the predicted full-length transcripts of *Rspe_dsx*, *Mnat_dsx*, *Csex_dsx1*, and *Csec_dsx2* was encoded by a single exon in their genome. Based on the transcriptome data in *R. speratus*^21^, *Rspe_dsx* was expressed only in male reproductives (primary kings), soldiers, and workers, but not in females, and in both heads (*t* = 3.99, *p* < 0.01, GLM) and other body parts (referred to as just “body”) (*t* = 10.97, *p* < 0.001, GLM) (Fig. 4A). Quantitative RT-PCR also showed male-specific expression patterns even in the nymphs (*t* = 5.69, *p* < 0.01, GLM, Fig. 4B) and eggs (*t* = 3.10, *p* < 0.05, GLM, Fig. 4C). In addition, *Ntak_dsx* was expressed only in males, regardless of caste (*t* = 3.70, *p* < 0.01, GLM, Fig. 4D). These results indicated that termite *dsx* was not alternatively spliced, but transcribed in a male-specific manner.

### Predicted binding sites and expression patterns of putative regulatory factors for male-specific transcription of *Rspe_dsx*

To examine the proximate mechanism for male-specific transcription of termite *dsx*, we searched for putative transcription factors that bind to the promoter region of *Rspe_dsx*, and those that are expressed in a male- or female-specific manner. First, 1 kb upstream of the transcription start site of *Rspe_dsx* was extracted as a transcriptional regulatory region, as defined by Toyota et al.^27^. Next, de novo motif discovery was performed for the promoter using hypergeometric optimisation of motif enrichment (HOMER) v4.11^28^, based on the equipped motif library of known transcription factors in insects. Although 32 putative TFBSs were detected in the promoter, 20 homologues out of the 32 transcription factors existed in the *Reticulitermes* genome, of which five were duplicated in the genome (Table 2). Based on the transcriptome data in *R. speratus*^21^, however, none of them were expressed in a sex-specific manner in either heads or bodies, except for the *vielfaltig* (*vfl*) orthologue (GLM, Table 2). The expression levels of the *vfl* orthologue were significantly different between sexes only in the bodies of reproductives, but similar patterns were not observed in other castes (Fig. S1).

**Table 2:** Transcription factors with putative binding sites detected in the *R. speratus* genome and their expression differences between sexes.

Gene symbol	Gene ID in *R. speratus*	FDR^a^ in heads	FDR^a^ in bodies^b^
*Trl*	RS014506	1.000	0.176
*grh*	RS004650	1.000	0.825
*Cf2-II*	RS004984	1.000	0.778
*ara*	RS009368	1.000	0.492
*Hr46*	RS006489	1.000	0.910
*br-Z4*	RS006084	1.000	0.970
*br-Z4*	RS006083	1.000	0.628
*brk*	RS014094	1.000	0.217
*br-Z3*	RS006084	1.000	0.970
*dve*	RS006834	1.000	0.836
*ttk*	RS007158	1.000	0.914
*ttk*	RS007159	1.000	0.931
*pros*	RS012493	1.000	0.890
*pros*	RS012492	1.000	0.217
*vfl*	RS003882	1.000	0.275 × 10^–3^
*ap*	RS004033	1.000	0.913
*ap*	RS004477	1.000	0.633
*D*	RS010190	1.000	0.822
*prd*	RS008463	1.000	0.967
*Hsf*	RS003326	1.000	0.406
*su(Hw)*	RS012357	1.000	0.791
*br-Z2*	RS006084	1.000	0.970
*br-Z2*	RS006083	1.000	0.628
*Pnt*	RS003359	1.000	0.807
*sd*	RS008357	1.000	0.919

^a^FDR based on GLM with sex using edgeR.

^b^Body: the thorax and abdomen.

## Discussion

**Figure 5:**
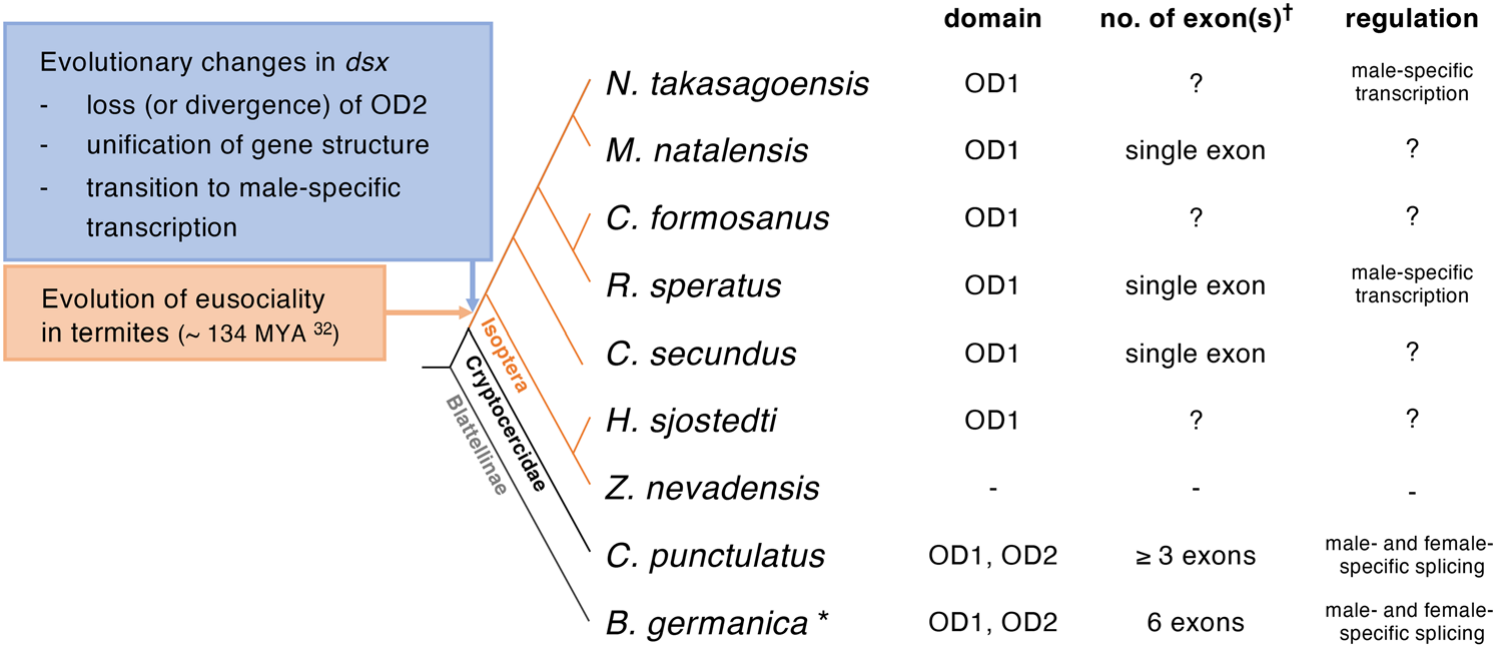
Evolutionary changes in *dsx* regulation associated with social evolution in termites. The presence of OD1 and OD2, numbers of exons containing coding sequences (^†^), and regulatory manner of *dsx* homologues were compared among the German cockroach, the subsocial woodroach, and seven termite species. “-” and “?” mean “not detected” and “unknown”, respectively. *Data on *B. germanica* were cited from Wexler et al.^9^. The tree topology was based on that of Bourguignon et al.^57^, Bucek et al.^31^, and Evangelista et al.^32^.

The *dsx* orthologue of the subsocial *Cryptocercus* woodroach was spliced in a sex-specific manner, similar to that of the gregarious *Blattella* cockroach and holometabolous insects (Fig. 5). Surprisingly, however, termite *dsx* homologues did not exhibit sex-specific splicing, and their transcription was regulated in a male-specific manner. Male- or female-biased expression of *dsx* has been reported in at least 8 species of non-insect arthropods, including water fleas^5,27^, red claw crayfish^29^, Chinese shrimp^30^ (Crustacea), and mite^7^ (Chelicerata), suggesting that sex-biased *dsx* transcriptions are an ancestral state in Arthropoda^9^. In contrast, *dsx* produces sex-specific splice isoforms in major holometabolous insect orders (Coleoptera, Hymenoptera, Lepidoptera, and Diptera) and those hemimetabolous insects examined previously^6,9,26^ except for the louse *Pediculus humanus* and the hemipteran *Bemisia tabaci.* These two hemimetabolous insects have *dsx* homologues with non-sex-specific isoforms^8,9^. Consequently, the sex-specific alternative splicing of *dsx* would have been acquired early in the evolution of insects, and secondarily lost in some hemimetabolous insects^9^, including termites. Within the cockroach and termite clade, after ancestral termites diverged from the common ancestor of subsocial *Cryptocercus* and eusocial termites (around 140.6 MYA, ranging from 112.6 to 170.5 MYA^31,32^) the data suggest that sex-specific *dsx* splicing was lost, and the male-specific *dsx* transcription was alternatively acquired in eusocial termites (Fig. 5). It remains unclear whether the *dsx* orthologue was actually lost in the genome of *Z. nevadensis*. However, it is interesting to note that *Z. nevadensis* has a genome half the size of other termites (562 Mb)^19^, suggesting the possibility that some genes may be missing. A further exhaustive search is needed.

Although *dsx* consists of approximately five exons in 145 species belonging to 39 holometabolous insect families^26^, two *dsx* paralogs (*dsx1* and *dsx2*) in water fleas consist of four and two exons, respectively^5^. Its homologue in Chinese shrimp consists of two exons^30^, and those in mites^7^ and termites consist of only a single exon (Fig. 5). Moreover, the present study showed no homologous OD2 in termite *dsx* (Fig. 5), suggesting that OD2 has indeed been lost or has diverged beyond our ability to detect it^6^. OD2 is conserved in *dsx* orthologues with sex-specific splicing in holometabolous insects, as well as in those with sex-specific transcription in water fleas^5,27^ and Chinese shrimp^30^ but not in red claw crayfish^29^. Both OD1 and OD2 are related to binding target DNA as a dimeric DNA binding unit in the fruit fly *D. melanogaster*: OD1 enables Dsx to bind target DNA and dimerizes weakly in a DNA-dependent manner^12^, whereas OD2 is necessary for dimerization and enhances DNA recognition of OD1^33^. In addition, fly OD2 contains sex-specific spliced sequences, which may be involved in the formation of sex-specific units for transcriptional regulation of downstream target genes through either its sex-specific interactions with the transcriptional machinery or its sex-specific DNA binding^12^. The decreasing numbers of *dsx* exons in some arthropods and termites and the loss (or divergence) of OD2 in termites would be associated with the evolution of *dsx* regulation, although it remains unknown whether such evolutionary changes in the domain and exon organisation were the cause or consequence of the loss of sex-specific splicing. Future studies that aim to characterize the *dsx* orthologue in *Mastotermes darwiniensis*, the earliest branching termite lineage, might help clarify the issue.

The sex determination cascade has never been examined in termites. Almost all orthologues of sex determination genes reported in holometabolous insects (e.g., *transformer*, *transformer-2*, and *Sex-lethal*) are conserved in the genome of *R. speratus*^24^. However, these orthologues encode splicing factors and are unlikely to directly regulate male-specific *dsx* transcription. Male-specific transcription should be regulated by a transcription factor expressed in a sex-specific manner in every caste, as shown in termite *dsx*. Although we found 27 putative TFBSs in the *Rspe_dsx* promoter region, no orthologues of 27 transcription factors showed sex-specific expression patterns similar to those of *Rspe_dsx*. However, our motif search was based on the binding motifs found in the fruit fly *D. melanogaster*. In addition, unidentified binding motifs could be present out of the transcriptional regulatory region examined in this study. Further investigations are needed to determine whether the unidentified transcription factors expressed in a sex-specific manner would regulate male-specific *dsx* transcription in termites.

Although *dsx* was sexually spliced in gregarious *Blattella* and subsocial *Cryptocercus* cockroaches, a single *dsx* transcript was expressed only in the male of eusocial lower and higher termites. Given that cooperative brood care by parents and a generational overlap was likely acquired in the last common ancestor of *Cryptocercus* woodroach and termites^32^, the transition in *dsx* regulation in termites is associated with the evolution of eusociality, especially the acquisition of reproductive division of labor (Fig. 5). Such association between *dsx* expression and social traits also has been suggested in ants. The *dsx* orthologues were sexually spliced in all 5 ants examined, whereas their *dsx* was expressed in a male-biased manner in 3 species with an environmental caste determination system, but not in 2 species with a genetic caste determination system^34^. Additionally, the OD2 was present in 2 basal ant species, but absent in 17 ants belonging to the subfamilies with more advanced social traits^34^.

The male-specific transcription of termite *dsx* would play a role in the regulation of reproductive division of labour, particularly in soldiers, the first sterile caste that evolved in Isoptera^35^. Some species, particularly in the crown termite family Termitidae, have sex-specific or sex-biased soldier ratios and soldier differentiation pathways^36^. For example, all soldiers are females in most species of the subfamilies Termitinae and Macrotermitinae, whereas all soldiers are males in most of the examined species in Nasutitermitinae. Soldier differentiation requires high juvenile hormone titres in workers, and the strongly biased soldier-sex ratio may be rooted in differences in juvenile hormone titres (and probably related gene expression levels) between male and female workers^37,38^. Reproductive caste differentiation is also regulated in a sex-specific manner (reviewed in Oguchi et al.^39^). “Queen genes” with high expression levels in female reproductives have been identified in some species (reviewed in Korb^40^), and in male reproductives upregulated genes likely associated with male fertility have been identified in species with available genome sequences^15,19,24^. Termite *dsx* could regulate this sexually dimorphic gene expression by upregulating or downregulating the expression of its target genes in a male-specific manner, resulting in reproductive and non-reproductive division of labour.

## Materials and methods

### Insects

Seven mature colonies of *R. speratu*s were collected in Toyama Prefecture in 2016 and 2019. One *H. sjostedti* mature colony was collected in Yakushima Island, Kagoshima Prefecture in 2015. Two *N. takasagoensis* mature colonies were found on Ishigakijima Island, Okinawa Prefecture, in 2017 and 2018. They were kept in the laboratory at ca. 25 °C in constant darkness. Three kin groups of *C. punctulatus* were collected at Mountain Lake Biological Station, Giles County, VA in April 2015–2017^41^ and kept at 15 °C in constant darkness. Testes and ovaries were dissected from three males and three females, respectively, and stored at − 80 °C until RNA extraction for subcloning and expression analyses of *Cpun_dsx*.

### BLAST searches for *dsx* and *dmrt* homologues in termites and cockroaches

tBlastX searches were performed against genome and transcriptome databases of six termite species and one *Cryptocercus* woodroach species (Table 1), using SequenceServer^42^. Either the OD1 (45 amino acids) or OD2 (45 amino acids) sequences of *B. germanica dsx* were used as queries. Sequences with an E value less than 10^–5^ were selected as candidates for *dsx* orthologues and used for phylogenetic analysis.

### Construction of phylogenetic tree of OD1 sequences and identification of *dsx* orthologues

To identify the *dsx* orthologue, a phylogenetic tree of OD1 sequences (135 bp with no gaps) was constructed according to previous studies^5,43,44^. We used 84 OD1-containing genes (Table S3 ‘OTU’). The DMRT7 genes of vertebrates (mouse and cattle) were used as the outgroups. Phylogenetic relationships were inferred using Bayesian inference (BI) and maximum likelihood (ML) methods. For BI, the most appropriate model of sequence evolution was determined using the model selection option implemented in MEGA version 7.0.21^45^, and the GTR + G model was selected. Using MrBayes version 3.2.6^46^, a total of 100,000 trees were obtained (ngen = 10,000,000, samplefreq = 100). The first 25% of these (25,000) were discarded as burn-ins, and a 50%-majority-rule consensus tree was produced. For ML, 1000 bootstrap replicates were performed based on the same model of sequence evolution as BI in MEGA 7.0.21, with the default tree inference options.

### Synteny analysis

According to Wexler et al.^9^, the synteny of *dsx* was examined based on the proximity to the transcription factor *prospero* (*pros*). The *dsx* locates at the genomic position close to *pros*, with an intervening distance between 17 and 245 kb in eight different insect orders (Blattodea, Orthoptera, Ephemeroptera, Phthiraptera, Thysanoptera, Hymenoptera, Coleoptera, and Lepidoptera)^9,23^. To identify the *pros* ortholog in each termite, tBlastX searches were performed against gene models of *R. speratus*^24^ and *M. natalensis*^18^ using SequenceServer^42^, and BlastX searches against predicted protein databases of *Cryptotermes secundus*^15^ and *Coptotermes formosanus*^22^. The *pros* sequence of *B. germanica* (Accession No. PYGN01000615) was used as a query. To show the genomic position, tBlastN searches then were performed against the genome database of *Cryptotermes secundus*^15^ and *Coptotermes formosanus*^22^, querying with the obtained Pros protein sequence in each species using SequenceServer^42^.

### Subcloning of the candidate *dsx* orthologues

Female and male alate reproductives (winged adults) of *R. speratus* were obtained from three colonies collected in 2016. According to previous studies^47,48^, incipient colonies were established using unrelated female and male alates. A total of 30 eggs was obtained, and 3 primary queens and 3 primary kings were randomly selected from 20 colonies at 1.5 months after colony establishment. Total RNA of all the eggs was extracted and treated with DNase I using the ReliaPrep RNA Tissue Miniprep System (Promega, USA). Total RNA of the primary queens or kings was extracted from the body (except for the head parts, three individuals of each sex) using ISOGEN II (Nippongene, Japan). Total RNA of female or male alates of *N. takasagoensis* (using the colony collected in 2017) was extracted from the body (except for the head parts, five individuals in each sex) using ISOGEN II. Total RNA from the secondary queen or king of *H. sjostedti* (using the colony collected in 2015) was extracted from the body (except for the head part, one individual in each sex) using ISOGEN II. An ovary and testis of *C. punctulatus* were dissected from a female adult and male adult, respectively. Total RNA was extracted from each of the gonads using ISOGEN I and II, and then treated with RNase-free DNase I (Takara, Japan). The purity and quantity of the extracted RNA were measured using a NanoVue spectrophotometer (GE Healthcare Bio-Sciences, Japan).

The 5′ and 3′ ends of *Cpun_dsx* were amplified using the SMARTer RACE 5′/3′kit (TaKaRa, Shiga, Japan) and gene-specific primers designed for OD2 (“*Cpun_dsx* OD2 5'RACE” and “*Cpun_dsx* OD2 3'RACE”, Table S1). Its male-specific exon was amplified using Advantage^®^ 2 Polymerase Mix (TaKaRa), the gene-specific primer “*Cpun_dsx* OD2 3'RACE”, and a reverse primer in the male-specific exon of *Blattella dsx*, which was located at terminal codon and 3′UTR (“*Cpun*_*dsx* male-specific exon-R”, Table S1). The 3′ ends of *Rspe_dsx*, *Hsjo_dsx*, and *Ntak_dsx* were also amplified using the SMARTer RACE 5′/3′ Kit and newly designed gene-specific primers (Table S1). The amplified fragments were subcloned into the pGEM-T easy vector system (Promega), and the nucleotide sequences of each fragment were determined using ABI Prism Big Dye Terminator v3.1 Cycle Sequencing Kit in conjunction with a 3500 Genetic Analyzer (Applied Biosystems, Foster City, CA, USA). The newly identified sequences of *Rspe_dsx*, *Ntak_dsx,* and *Hsjo_dsx* were deposited in the GenBank/EMBL/DDBJ database under accession numbers: LC635717, LC635718, and LC635719 (Table 1).

### RNA-seq analysis in *Reticulitermes speratus*

RNA-seq data of *R. speratus* were used to compare the expression levels of *Rspe_dsx* (Table 1) among three castes (workers, soldiers, and reproductives), two body parts (heads and the remaining parts), and two sexes (biological triplicates; NCBI BioProject Accession No. PRJDB5589^21^). The filtered RNA-seq reads were mapped onto their genome assembly^20^ using TopHat v2.1.021. Transcript abundances were then estimated using the featureCounts program of the Subread package^49^. To compare gene expression levels among castes and between sexes, first, counts per million (CPM) were calculated from the estimated transcript abundances. Genes with at least CPM of 1 in at least three samples were kept for subsequent analyses. CPM values were then normalized with the trimmed mean of M-values (TMM) algorithm in edgeR^50^. Differential gene expression analyses were performed separately for each body part using a GLM with two factors, namely, caste and sex implemented in edgeR, and then, genes with a false discovery rate (FDR) < 0.01 were identified as genes expressed in a sex-specific manner. RPKM (Reads Per Kilobase Million) values were calculated by dividing the CPM values by the length of the genes in kilobases.

### Real-time quantitative PCR

Gene-specific primers were designed against *Rspe_dsx*, *Ntak_dsx,* and *Cpun_dsx* using Primer3Plus^51^ for real-time quantitative PCR (Table S2). Total RNA of female and male nymphs of *R. speratus* (from the colony collected in 2019) was individually extracted from whole bodies (10 individuals in each sex) using ISOGEN II. Total RNA of male (minor and medium) workers, female (medium and major) workers, and male soldiers of *N. takasagoensis* (from the colony collected in 2018) was extracted from the whole body of five separate individuals using ISOGEN II. Total RNAs derived from the gonads of adult woodroaches were also used. DNase treatment was performed using the same method described above. According to previous studies^47,48^, incipient colonies (queen-king and two-queen colonies) of *R. speratus* were established using alates derived from three colonies collected in 2019. Eggs produced sexually in queen-king colonies and asexually in queen-queen colonies develop into both sexes and only females, respectively. Total RNA was extracted from 30 sexual and 30 asexual eggs (triplicated) and treated with DNase I using ReliaPrep RNA Tissue Miniprep System (Promega). cDNAs were synthesised from the purified RNA using a High-Capacity cDNA Reverse Transcription Kit (Applied Biosystems). Biological replications (n = 3–5) were prepared for each category. Expression analyses were performed using Thunderbird SYBR qPCR Mix (Toyobo, Japan) with a MiniOpticon Real-time PCR system (Bio-Rad, Japan) and an Applied Biosystems 7500 Fast Real-Time PCR System (Applied Biosystems).

To determine a sustainable internal control gene for *R. speratus*, the expression levels of six genes [*EF1-α* (accession no. AB602838), *NADH-dh* (no. AB602837), *β-actin* (no. AB520714), *GstD1* (gene id RS001168), *EIF-1* (RS005199), and *RPS18* (RS015150)] were evaluated using the GeNorm^52^ and NormFinder^53^ software (Table S4). For *C. punctulatus* and *N. takasagoensis*, according to previous studies^41,54^, three genes [*β-actin* (nos. Cp_TR19468 and AB501107), *NADH-dh* (Cp_TR49774 and AB50119), and *EF1-α* (AFK49795 and AB501108)] were evaluated (Tables S5 and S6). All gene-specific primers were designed using Primer3Plus (Table S2). We confirmed the amplification of a single PCR product using dissociation curves and product sizes.

The expression levels were statistically analysed using GLMs with gamma error distribution and log link function. The fixed effects were sex, caste, and their interaction. Multiple comparisons were performed using general linear hypothesis testing (glht function) with Tukey adjustment in the “multcomp” R package^55^. These analyses were conducted using R ver. 4.0.3 (available at http://cran.r-project.org/).

### Search for transcription factor binding motif

The *Reticulitermes* genome was loaded into the database of HOMER v4.11 using the “loagGenome.pl” program. The “findMotifsGenome.pl” program was executed to search for *Drosophila* motif collections using the “-mcheck” option against the promoter of *Rspe_dsx*, which was the 1.0 kbp upstream region from its transcription start site. The nucleotide sequences of *Drosophila* transcription factors that were detected in their binding sites were subjected to a BlastX search for orthologues against the protein database of *R. speratus*^41^ using BLAST + (2.10.1)^56^. The expression levels of these orthologues were extracted from their transcriptome data^21^ and statistically analysed using GLMs with gamma error distribution and log link function, as mentioned above.

## Supplementary Information

Supplementary Information.
