# Publisher Correction: Evolutionary transition of *doublesex* regulation from sex-specific splicing to male-specific transcription in termites

**DOI:** 10.1038/s41598-021-97591-y

**Published:** 2021-09-07

**Authors:** Satoshi Miyazaki, Kokuto Fujiwara, Keima Kai, Yudai Masuoka, Hiroki Gotoh, Teruyuki Niimi, Yoshinobu Hayashi, Shuji Shigenobu, Kiyoto Maekawa

**Affiliations:** 1 Graduate School of Agriculture, Tamagawa University, Machida, Tokyo 194-8610 Japan; 2 Graduate School of Science and Engineering, University of Toyama, Gofuku, Toyama 930-8555 Japan; 3 Institute of Agrobiological Sciences, NARO (National Agriculture and Food Research Organization), Tsukuba, Ibaraki 305-8634 Japan; 4 Department of Biological Science, Faculty of Science, Shizuoka University, Suruga-ku, Shizuoka, 422-8529 Japan; 5 Division of Evolutionary Developmental Biology, National Institute for Basic Biology, Okazaki, Aichi 444-8585 Japan; 6 Department of Basic Biology, School of Life Science, The Graduate University for Advanced Studies, SOKENDAI, Okazaki, Aichi 444-8585 Japan; 7 Department of Biology, Keio University, Yokohama, Kanagawa 223-8521 Japan; 8 NIBB Research Core Facilities, National Institute for Basic Biology, Okazaki, Aichi 444-8585 Japan; 9 Faculty of Science, Academic Assembly, University of Toyama, Gofuku, Toyama 930-8555 Japan

Correction to: *Scientific Reports* 10.1038/s41598-021-95423-7, published online 06 August 2021

The original version of this Article contained an error in Figure 2, where the asterisks were incorrectly displayed as exclamation marks. The original Figure 2 and accompanying legend appear below.

**Figure 2:**
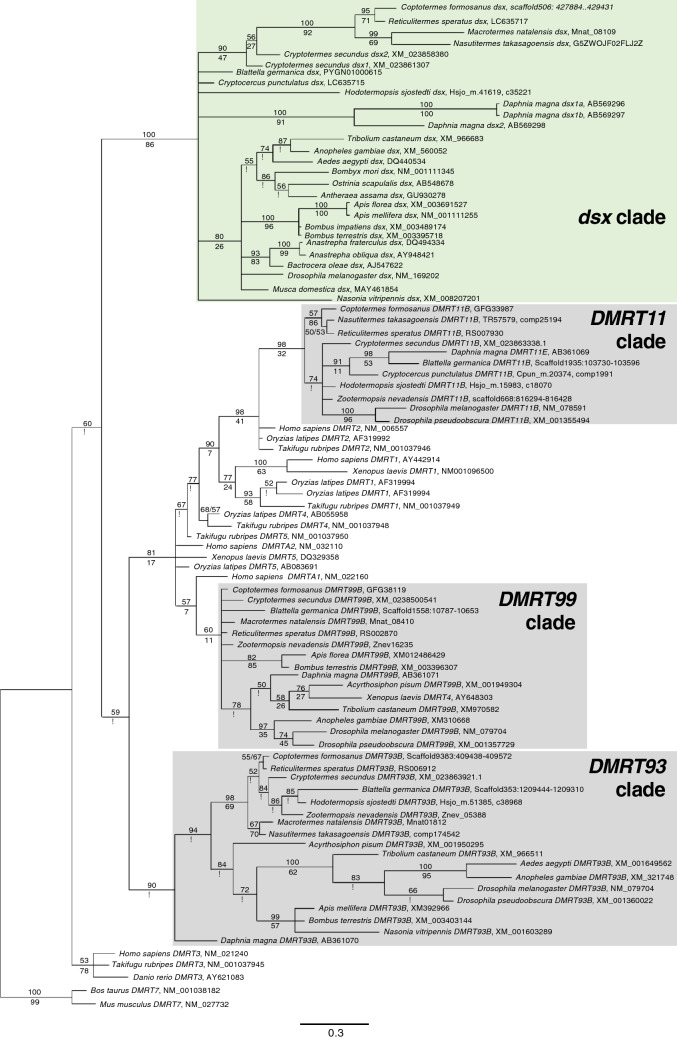
Molecular phylogenetic tree of *dsx* and *DMRT* homologues. Bayesian tree of *dsx* and *DMRT* of insects and crustaceans was constructed based on the OD1 sequences (135 bp with no gaps). Numbers shown above branches represent the Bayes posterior probabilities. Bootstrap values (1000 replicates) are shown below branches to indicate the level of support in the ML method. An asterisk indicates that a node is not supported by the ML method.

The original Article has been corrected.

