# Supplementary material for: Evolutionary transition of doublesex regulation from sex-specific splicing to male-specific transcription in termites
Source: Sci Rep. 2021 Aug 6;11:15992. doi: 10.1038/s41598-021-95423-7 (PMC8346542; doi:10.1038/s41598-021-95423-7)
Supplement: Supplementary file 1 — Supplementary Information. [file 41598_2021_95423_MOESM1_ESM.pdf]

Supplementary Information for:

Evolutionary transition of *doublesex* regulation from sex-specific splicing to male-specific transcription in termites

Satoshi Miyazaki[1], Kokuto Fujiwara[2], Keima Kai[2], Yudai Masuoka[2,3], Hiroki Gotoh[4], Teruyuki Niimi[5,6], Yoshinobu Hayashi[7], Shuji Shigenobu[6,8] Kiyoto Maekawa[9]

[1] Graduate School of Agriculture, Tamagawa University, Machida, Tokyo 194-8610, Japan

[2] Graduate School of Science and Engineering, University of Toyama, Gofuku, Toyama, 930-8555, Japan

[3] Institute of Agrobiological Sciences, NARO (National Agriculture and Food Research Organization), Tsukuba, Ibaraki 305-8634, Japan

[4] Department of Biological Science, Faculty of Science, Shizuoka University, Suruga-ku, Shizuoka, 422-8529, Japan

[5] Division of Evolutionary Developmental Biology, National Institute for Basic Biology, Okazaki, Aichi, 444-8585 Japan

[6] Department of Basic Biology, School of Life Science, The Graduate University for Advanced Studies, SOKENDAI, Okazaki, Aichi, 444-8585 Japan

[7] Department of Biology, Keio University, Yokohama, Kanagawa, 223-8521, Japan

[8] NIBB Research Core Facilities, National Institute for Basic Biology, Okazaki, Aichi, 444-8585 Japan

[9] Faculty of Science, Academic Assembly, University of Toyama, Gofuku, Toyama, 930-8555, Japan

Table S1. Primers used for RACE PCRs and subcloning.

species	primer name	primer sequence
Cryptocercus punctulatus	*Cpun_dsx* OD2 3'RACE	5'-CCCTTCCGCTCATCTATGTTGTGCTTC-3'
Cryptocercus punctulatus	*Cpun_dsx* OD2 5'RACE	5'-AGCACAACATAGATGAGCGGAAGGGCTT-3'
Cryptocercus punctulatus	*Cpun_dsx* male-specific exon-R	5'- GGAGGTGGAAGGTCACTGCTA -3'
Hodotermopsis sjostedti	*Hsjo_dsx* OD2 3'RACE	5'-GGAAGTGAACCATCTCAAAGGCCACA-3'
Reticulitermes speratus	*Rspe_dsx* OD2 3'RACE	5'-CCAACCTGTGCCCGGTGTCGAAATCATT-3'
Nasutitermes takasagoensis	*Ntak_dsx* OD2 3'RACE	5'-GCCCGGTGTCGAATTCATAAGGAACTAA-3'

Table S2. Primers used for quantitative RT-PCRs.

species	primer name	prime sequence
Cryptocercus punctulatus	*Cpun_dsx* qPCR-F	5'- TGTGCTTCAGGTCTCACAATCG -3'
Cryptocercus punctulatus	*Cpun_dsx* qPCR-Rf	5'- TGAGTTCATAGCAAATGAATACATGTAATGT -3'
Cryptocercus punctulatus	*Cpun_dsx* qPCR-Rm	5'- CCATGGACCGCAGCTGTT -3'
Cryptocercus punctulatus	*Cpun_beta-actin* qPCR-F	5'- TTCCTGGGTATGGAATCCTG -3' *
Cryptocercus punctulatus	*Cpun_beta-actin* qPCR-R	5'- GTGTTGGCGTACAGGTCCTT -3' *
Cryptocercus punctulatus	*Cpun_EF-1alpha* qPCR-F	5'- CCTGGGCACAGAGATTTCAT -3' *
Cryptocercus punctulatus	*Cpun_EF-1alpha* qPCR-R	5'- GAACTCTCCCGTACCAGCAG -3' *
Cryptocercus punctulatus	*Cpun_NADH-dh* qPCR-F	5'- CGGTTTGAAATTGTTTTTATGGA -3' *
Cryptocercus punctulatus	*Cpun_NADH-dh* qPCR-R	5'- AAATTAGCACCCAACCCTGA -3' *
Reticulitermes speratus	*Rspe_dsx* qPCR-F	5'- ACCTCCGTACCAAACCAACCTG -3'
Reticulitermes speratus	*Rspe_dsx* qPCR-R	5'- TTACCGCCCTCCGCTACAAC -3'
Reticulitermes speratus	*Rspe_beta-actin* qPCR-F	5'- AGCGGGAAATCGTCCGTGA -3'
Reticulitermes speratus	*Rspe_beta-actin* qPCR-R	5'- CAATGGTGATGACCTGCCCAT -3'
Reticulitermes speratus	*Rspe_EF1-alpha* qPCR-F	5'- GGTGATGCGGCTATTGTTAACC -3'
Reticulitermes speratus	*Rspe_EF1-alpha* qPCR-R	5'- GTGGTGGGAATTCTGAGAAAGATT -3'
Reticulitermes speratus	*Rspe_NADH-dh* qPCR-F	5'- GCTGGGGGGGTTATTCATTCCAT -3'
Reticulitermes speratus	*Rspe_NADH-dh* qPCR-R	5'- GGCATACCACAAAGGGCAAAA -3'
Reticulitermes speratus	*Rspe_GstD1* qPCR-F	5'- GCTGTTGGTGTGGATTTGAA -3'
Reticulitermes speratus	*Rspe_GstD1* qPCR-R	5'- GTATGCTGCGGGTTCATCTT -3'
Reticulitermes speratus	*Rspe_EIF-1* qPCR-F	5'- ATGGTAGGCTTGAAGCGATG -3'
Reticulitermes speratus	*Rspe_EIF-1* qPCR-R	5'- TTTGCATCCTGGTAGTCACG -3'
Reticulitermes speratus	*Rspe_RPS18* qPCR-F	5'- ACTCTCAGCTCACATCCAGT -3'
Reticulitermes speratus	*Rspe_RPS18* qPCR-R	5'- CCTCAGGCCCCAATAATGTC -3'
Nasutitermes takasagoensis	*Ntak_dsx* qPCR-F	5'- TAAAGGCCGTACCACACCAA -3'
Nasutitermes takasagoensis	*Ntak_dsx* qPCR-R	5'- ACAATCGCGACAATGACACT -3'
Nasutitermes takasagoensis	*Ntak_beta-actin qPCR-F*	5'- AGCGGGAAATCGTACGTGAC -3' †
Nasutitermes takasagoensis	*Ntak_beta-actin qPCR-R*	5'- CAATGGTGATGACCTGGCCAT -3' †
Nasutitermes takasagoensis	*Ntak_EF1-alpha qPCR-F*	5'- GGTGATGCGCTATTGTTAACC -3' †
Nasutitermes takasagoensis	*Ntak_EF1-alpha qPCR-R*	5'- GTGGTGGGAATTCTGAGAAAGATT -3' †
Nasutitermes takasagoensis	*Ntak_NADH-dh qPCR-F*	5'- GCTGGGGGCGTTATTCATTCTA -3' †
Nasutitermes takasagoensis	*Ntak_NADH-dh qPCR-R*	5'- GGCATGCCACAAAGAGCAAAA -3' †

* from Masuoka et al.[41], † from Hojo et al.[58]

Table S3. Information of OTU used for phylogenetic analysis.

Clade	Gene	Taxon	Organism	DNA accession No.
dsx	*dsx*	Blattodea	*Blattella germanica*	PYGN01000615
	Cpun_dsx	Blattodea	*Cryptocercus punctulatus*	LC635715
	Cfor_dsx	Isoptera	*Coptotermes formosanus*	scaffold506: 427884..429431
	Csec_dsx1	Isoptera	*Cryptotermes secundus*	XM_023861307
	Csec_dsx2	Isoptera	*Cryptotermes secundus*	XM_023858380
	Hsjo_dsx	Isoptera	*Hodotermopsis sjostedti*	Hsjo_m.41619, c35221
	Mnat_dsx	Isoptera	*Macrotermes natalensis*	Mnat_08109
	Ntak_dsx	Isoptera	*Nasutitermes takasagoensis*	G5ZWOJF02FLJ2Z
	Rspe_dsx	Isoptera	*Reticulitermes speratus*	LC635717
	dsx1a	Crustacea: Diplostraca	*Daphnia magna*	AB569296
	dsx1b	Crustacea: Diplostraca	*Daphnia magna*	AB569297
	dsx2	Crustacea: Diplostraca	*Daphnia magna*	AB569298
	dsx	Coleoptera	*Tribolium castaneum*	XM_966683
	dsx	Hymenoptera	*Apis florea*	XM_003691527
	dsx	Hymenoptera	*Apis mellifera*	NM_001111255
	dsx	Hymenoptera	*Bombus impatiens*	XM_003489174
	dsx	Hymenoptera	*Bombus terrestris*	XM_003395718
	dsx	Hymenoptera	*Nasonia vitripennis*	XM_008207201
	dsx	Lepidoptera	*Bombyx mori*	NM_001111345
	dsx	Lepidoptera	*Ostrinia scapulalis*	AB548678
	dsx	Diptera	*Aedes aegypti*	DQ440534
	dsx	Diptera	*Anastrepha fraterculus*	DQ494334
	dsx	Diptera	*Anastrepha obliqua*	AY948421
	dsx	Diptera	*Anopheles gambiae*	XM_560052
	dsx	Diptera	*Antheraea assama*	GU930278
	dsx	Diptera	*Bactrocera oleae*	AJ547622
	dsx	Diptera	*Drosophila melanogaster*	NM_169202
	dsx	Diptera	*Musca domestica*	AY461854
DMRT11	*DMRT11E*	Crustacea: Diplostraca	*Daphnia magna*	AB361069
	DMRT11B	Blattodea	*Blattella germanica*	Bger_genome_scaffold:Scaffold1935:103730-103596
	DMRT11B	Blattodea	*Cryptocercus punctulatus*	Cpun_m.20374, comp1991
	DMRT11B	Isoptera	*Hodotermopsis sjostedti*	Hsjo_m.15983, c18070
	DMRT11B	Isoptera	*Zootermopsis nevadensis*	scaffold668:816294-816428
	DMRT11B	Isoptera	*Nasutitermes takasagoensis*	TR57579, comp25194
	DMRT11B	Isoptera	*Cryptotermes secundus*	XM_023863338.1
	DMRT11B	Isoptera	*Reticulitermes speratus*	RS007930
	DMRT11B	Isoptera	*Coptotermes formosanus*	GFG33987
	DMRT11E	Diptera	*Drosophila melanogaster*	NM_078591
	DMRT11E	Diptera	*Drosophila pseudoobscura*	XM_001355494
DMRT93	*DMRT93B*	Crustacea: Diplostraca	*Daphnia magna*	AB361070
	DMRT93B	Blattodea	*Blattella germanica*	Bger_genome_scaffold:Scaffold353:1209444-1209310
	DMRT93B	Isoptera	*Hodotermopsis sjostedti*	Hsjo_m.51385, c38968
	DMRT93B	Isoptera	*Macrotermes natalensis*	Mnat_01812
	DMRT93B	Isoptera	*Nasutitermes takasagoensis*	comp174542
	DMRT93B	Isoptera	*Cryptotermes secundus*	XM_023863921.1
	DMRT93B	Isoptera	*Reticulitermes speratus*	RS006912
	DMRT93B	Isoptera	*Coptotermes formosanus*	Scaffold9383:409438-409572
	DMRT93B	Isoptera	*Zootermopsis nevadensis*	Znev_05388
	DMRT93B	Hemiptera	*Acyrthosiphon pisum*	XM_001950295
	DMRT93B	Coleoptera	*Tribolium castaneum*	XM_966511
	DMRT93B	Hymenoptera	*Apis mellifera*	XM_392966
	DMRT93B	Hymenoptera	*Bombus terrestris*	XM_003403144
	DMRT93B	Hymenoptera	*Nasonia vitripennis*	XM_001603289
	DMRT93B	Diptera	*Aedes aegypti*	XM_001649562
	DMRT93B	Diptera	*Anopheles gambiae*	XM_321748
	DMRT93B	Diptera	*Drosophila melanogaster*	NM_079704
	DMRT93B	Diptera	*Drosophila pseudoobscura*	XM_001360022
DMRT99	*DMRT99B*	Crustacea: Diplostraca	*Daphnia magna*	AB361071
	DMRT99B	Blattodea	*Blattella germanica*	Bger_genome_scaffold:Scaffold1558:10787-10653
	DMRT99B	Isoptera	*Coptotermes formosanus*	GFG38119
	DMRT99B	Isoptera	*Cryptotermes secundus*	XM_0238500541
	DMRT99B	Isoptera	*Macrotermes natalensis*	Mnat_08410
	DMRT99B	Isoptera	*Reticulitermes speratus*	RS002870
	DMRT99B	Isoptera	*Zootermopsis nevadensis*	Znev_16235
	DMRT99B	Hemiptera	*Acyrthosiphon pisum*	XM_001949304
	DMRT99B	Coleoptera	*Tribolium castaneum*	XM_970582
	DMRT99B	Hymenoptera	*Apis florea*	XM_012486429
	DMRT99B	Hymenoptera	*Bombus terrestris*	XM_003396307
	DMRT99B	Diptera	*Anopheles gambiae*	XM_310668
	DMRT99B	Diptera	*Drosophila melanogaster*	NM_079704
	DMRT99B	Diptera	*Drosophila pseudoobscura*	XM_001357729
DMRT1	*DMRT1*	Vertebrate: Primate	*Homo sapiens*	AY442914
	DMRT1	Vertebrate: Anura	*Xenopus laevis*	NM_001096500
	DMRT1	Vertebrate: Beloniformes	*Oryzias latipes*	AF319994
	DMY	Vertebrate: Beloniformes	*Oryzias latipes*	AB071534
	DMRT1	Vertebrate: Tetraodontiformes	*Takifugu rubripes*	NM_001037949
DMRT2	*DMRT2*	Vertebrate: Primate	*Homo sapiens*	NM_006557
	DMRT2	Vertebrate: Beloniformes	*Oryzias latipes*	AF319992
	DMRT2	Vertebrate: Tetraodontiformes	*Takifugu rubripes*	NM_001037946
DMRT3	*DMRT3*	Vertebrate: Primate	*Homo sapiens*	NM_021240
	DMRT3	Vertebrate: Tetraodontiformes	*Takifugu rubripes*	NM_001037945
	DMRT3	Vertebrate: Cypriniformes	*Danio rerio*	AY621083
DMRT4	*DMRTA1*	Vertebrate: Primate	*Homo sapiens*	NM_022160
	DMRT4	Vertebrate: Anura	*Xenopus laevis*	AY648303
	DMRT4	Vertebrate: Beloniformes	*Oryzias latipes*	AB055958
	DMRT4	Vertebrate: Tetraodontiformes	*Takifugu rubripes*	NM_001037948
DMRT5	*DMRTA2*	Vertebrate: Primate	*Homo sapiens*	NM_032110
	DMRT5	Vertebrate: Anura	*Xenopus laevis*	DQ329358
	DMRT5	Vertebrate: Beloniformes	*Oryzias latipes*	AB083691
	DMRT5	Vertebrate: Tetraodontiformes	*Takifugu rubripes*	NM_001037950
DMRT7	*DMRT7*	Vertebrate: Glires	*Mus musculus*	NM_027732
	DMRT7	Vertebrate: Cetartiodactyla	*Bos taurus*	NM_001038182

Table S4. Stability values of internal control gene candidates of *R. speratus* using GeNorm and NormFinder.

	GeNorm (stability value)	NormFinder (stability value)
Rspe_beta-actin	0.385	0.264
Rspe_EF1-alpha	0.246	0.106
Rspe_NADH-dh *	0.148	0.051
Rspe_GstD1	0.158	0.089
Rspe_EIF-1	0.148	0.061
Rspe_RPS18	0.161	0.093

* *Rspe_NADH-dh* was selected by GeNorm and NormFinder due to the lowest stability values.

Table S5. Stability values of internal control gene candidates of *C. punctulatus* using GeNorm and NormFinder.

	GeNorm (stability value)	NormFinder (stability value)
Cpun_beta-actin *	2.365	0.621
Cpun_EF1-alpha	2.665	1.306
Cpun_NADH-dh	3.237	2.075

* *Cpun_beta-actin* was selected by GeNorm and NormFinder due to the lowest stability values.

Table S6. Stability values of internal control gene candidates of *N. takasagoensis* using GeNorm and NormFinder.

	GeNorm (stability value)	NormFinder (stability value)
Ntak_beta-actin	0.908	0.505
Ntak_EF1-alpha *	0.757	0.237
Ntak_NADH-dh	0.983	0.603

* *Ntak_EF1-alpha* was selected by GeNorm and NormFinder due to the lowest stability values.

Figure S1

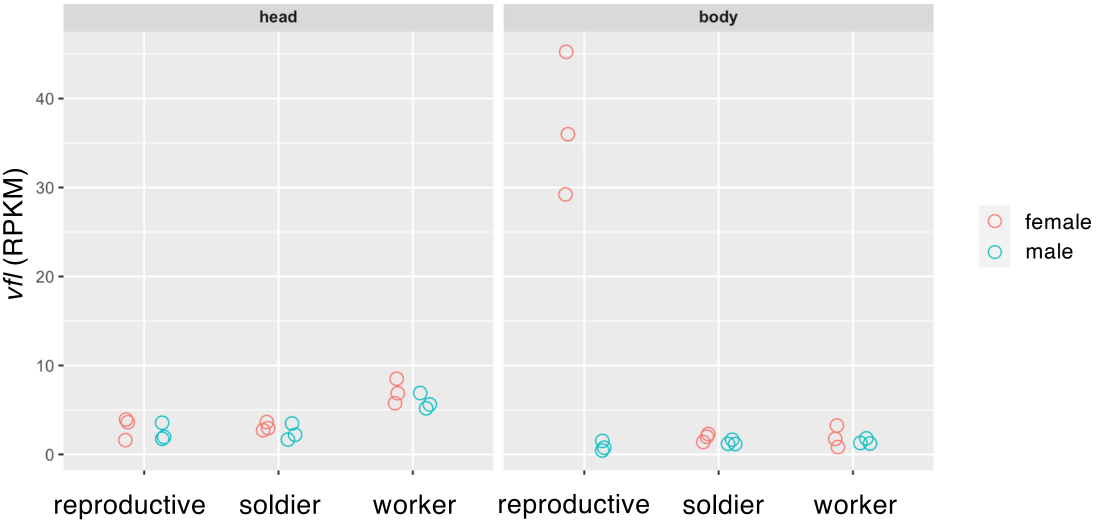

Figure S1. RPKM of *vfl* orthologue in each sex and in each caste of *R. speratus*; RNA-seq data were deposited in NCBI (DRA010978, biological triplicates [21]). The effects of sex, caste, and their interaction on gene expression levels were evaluated using GLM.

References

[58] Hojo, M., Toga, K., Itai, I. & Maekawa, K. Reference genes for real-time quantitative reverse transcriptase-PCR in the higher termite *Nasutitermes takasagoensis* (Isoptera: Termitidae) comparing soldiers with minor workers. Sociobiology 54, 509-520 (2009).
